# Social relevance of microbiology literacy

**DOI:** 10.1093/femsle/fnag051

**Published:** 2026-04-25

**Authors:** Juan L Ramos, Sukanya Lal, Utkarsh Sood, Princy Hira, Abhilash Kumar, Víctor de Lorenzo, Zulema Udaondo, Christin Baumgärtel, Lars M Blank, Julia Lorke, Nicole Maaßen, Max Chavarria, Kenneth N Timmis, Rup Lal

**Affiliations:** Estación Experimental del Zaidín, CSIC, 18008 Granada, Spain; PhiXGen Private Limited, Gurugram, Haryana 122001, India; Molecular Omics and Biotechnology Laboratory, Department of Zoology, Kirori Mal College, University of Delhi, Delhi 110007, India; Department of Zoology, Maitreyi College, University of Delhi, Delhi 110021, India; Molecular Biology and Genomics Research Laboratory, Department of Zoology, Ramjas College, University of Delhi, Delhi 110007, India; National Center of Biotechnology, CSIC, Cantoblanco, 28049 Madrid, Spain; National Center of Biotechnology, CSIC, Cantoblanco, 28049 Madrid, Spain; Dresden University of Technology, Chair of General Microbiology, 01062 Dresden, Germany; RWTH Aachen University, 52062 Aachen, Germany; RWTH Aachen University, 52062 Aachen, Germany; RWTH Aachen University, 52062 Aachen, Germany; Escuela de Química & Centro de Investigaciones en Productos Naturales (CIPRONA), Universidad de Costa Rica, 11501–2060 San José, Costa Rica; Centro Nacional de Innovaciones Biotecnológicas (CENIBiot), CeNAT-CONARE, 1174–1200 San José, Costa Rica; Technical University of Braunschweig, 38106 Braunschsweig, Germany; Acharya Narendra Dev College, University of Delhi, Delhi 110019, India

**Keywords:** Education, Microbiolology Literacy, Sustainability, science communicators, planetary health, global challenges

## Abstract

This Correspondence summarizes the session *“From Petri Dish to Planet Earth: Microbiology by All*” at FEMS MICRO Milan 2025 highlighting the urgent need for microbial literacy and societal engagement with microbiology. Chaired by Rup Lal and Juan-Luis Ramos, the session convened scientists, educators, and communicators to discuss strategies for promoting microbiology among communities, policymakers, and students. Central contributions focused on the International Microbiology Literacy Initiative (IMiLI), emphasizing global collaboration, intergenerational dialogue, and community-centered educational tools. Discussions showcased practical examples of microbial applications for sustainability and public engagement. The session conclusions, presented by Princy Hira, reaffirmed IMiLI’s mission to expand microbial literacy across borders and generations, recognizing educators as key drivers and science communicators as essential outreach ambassadors

## Teaser

Recognizing the crucial role of microbes in planetary and human and health empowers citizens to make informed knowledge-based decisions and engage in the shared stewardship of our planet.

## Microbes and global sustainability

The session served as an interdisciplinary forum on the roles of microbes (bacteria, algae, fungi, protozoans, archaea and viruses) in health, environment, agriculture, and climate resilience, alongside strategies for societal engagement. Rup Lal opened with a keynote on “*International Microbiology Literacy Initiative (IMiLI): Meeting the Need for Microbial Literacy and Promoting Microbiology among Communities, Policy Makers, Administrators, and School Children through a Worldwide Network of Microbiologists*.” He emphasized microbes as fundamental to planetary and human health and outlining IMiLI’s global network approach to outreach, education, and policy engagement, particularly through IMILI-SAC (International Microbiology Literacy Initiative- South Asian Centre, Delhi). He highlighted microbes as drivers of biogeochemical cycles, ecosystems stability, soil fertility, water quality, and climate regulation, noting their historical and ongoing role in oxygen production and the formation of the ozone layer that enabled terrestrial life. Today, photosynthetic microbes still generate about 50% of the oxygen we breathe (Crowther et al. 2024, Robador et al. 2024)

Juan-Luis Ramos outline “*IMiLI's Global Vision*” involving over 700 scientists and four regional centers. He emphasized the availability of free educational resources, the importance of intergenerational dialogue, and alignment with the United Nations Sustainable Development Goals (UN-SDGs), reaffirming the value of inclusive scientific communities for effective science communication (Timmis et al. 2019). He presented a suite of materials aimed at the general public, including Topic Framework lessons (TFs), Galleries of notable microorganisms and scientists presented in short, highly engaging articles, as well as excursions and experiments. All materials are freely accessible. (https://imili.org/)

Victor de Lorenzo presented “*Microbes Put in Action for a Better Environment*,”, addressing environmental challenges of microbial biotechnology, such as pollution mitigation, sustainable agriculture, and ecological restoration, sparking an extensive debate on the use and regulation of Genetically Manipulated Microorganisms for removal of toxic and recalcitrant chemicals.

Representing the IMiLI-South Asia Centre (IMiLI-SAC), Sukanya Lal discussed “*Workshops, Outreach, and Youth Engagement*” highlighting community engagement, school integration and public discourse, while emphasizing capacity building, hands-on learning, and sustained partnerships to nurture scientifically informed and socially responsible future generations. Princy Hira presented innovative educational tools developed at IMiLI-SAC, including visual modules, interactive activities, and story-based teaching strategies aimed at making microbiology engaging and accessible to schoolchildren. Particular emphasis was placed on *propagating microbial literacy* through creative educational tools such as flashcards, which simplify complex microbial concepts and visually communicate the multifaceted roles of microbes in achieving the United Nations Sustainable Development Goals (UN-SDGs). Each flashcard illustrated a specific SDG, showcasing how microbial communities contribute to its achievement. Utkarsh Sood complemented this with “*Developing and Disseminating Teaching Resources*”, focusing on the development of local teaching resources to integrate microbial literacy in schools and communities, thereby promoting ecological awareness, resilience, and community-based understanding. The interactive format transformed the session into a dialogue rather than a lecture, empowering participants to become *ambassadors of microbial literacy* capable of communicating the societal relevance of microbiology to diverse audiences and promoting science-informed action for sustainability. Julia Lorke further reinforced the importance of structured science education in fostering microbial literacy and nurturing scientifically informed communities.

## Microbial biotechnology and global challenges (UN-SDGs)

A roundtable at the World Café examined how microbes provide essential services, such as food production, medicine, water purification. Participants noted that most microbial diversity remains unexplored, while advances in genomics, systems and synthetic biology offer new opportunities for addressing global challenges (Crowther et al. 2024).

Despite their importance, microbes are often underrepresented in policy discussions, underscoring the need to strengthen links between microbial science, society, and decision-makers.

### The need for microbiology literacy

A second interactive roundtable addressed the “invisibility” of microbes and their frequent association with disease. Participants emphasized the need to present microbes as “tools” for solving global challenges, and enabling informed decision-making. Examples discussed included antibiotic resistance, vaccine hesitancy, allergies increases linked to over-sanitation and soil degradation (Anand et al. 2023, Timmis et al. 2024).

This World Café highlighted a key element of IMiLI, which seeks to address these gaps by linking microbes to everyday life and sustainability through culturally adapted, experience-based educational materials through a global network. Participants also emphasized the importance of regional collaboration and translation of materials to broaden impact.

### Beyond IMILI

Complementary initiatives further advance microbial literacy. The MicroMundo project combines citizen science with education, and address antimicrobial resistance (AMR) and support antibiotic discovery (Gil-Serna et al. 2025). Service-Learning (S-L) programs connect microbiology education with societal needs promoting health awareness and social responsibility (Valderrama et al. 2025). Additionally, integration of microbiology with art enhances engagement by making complex concepts more accessible and appealing (Yarzabal-Rodríguez and Batista-García 2025).

## Conclusion

The session underscored educators and science communicators as pivotal ambassadors of microbial understanding. Microbial literacy is a societal necessity rather than an academic luxury, essential for recognizing the role of microbes in sustaining life on Earth and addressing global challenges. Expanding microbial literacy across generations is critical to fostering a sustainable and resilient future. Microbiology should be embedded as a core component of basic education.
